# State of affairs of hybrid imaging in Europe: two multi-national surveys from 2017

**DOI:** 10.1186/s13244-019-0741-7

**Published:** 2019-05-21

**Authors:** Sergios Gatidis, Thomas Beyer, Minerva Becker, Katrine Riklund, Konstantin Nikolaou, Clemens Cyran, Christina Pfannenberg

**Affiliations:** 1 Department of Radiology, University Hospital Tübingen, 72076 Tübingen, Germany; 2 QIMP Team, Center for Medical Physics and Biomedical Engineering, Medical University Vienna, Vienna, Austria; 3 Department of Radiology and Medical Informatics, University Hospital Geneva, Geneva, Switzerland; 4 Department of Radiology, Radiation Sciences, Umeå University, 901 85 Umeå, Sweden; 5 Department of Radiology, University Hospital, LMU Munich, Marchioninistr. 15, 81377 Munich, Germany

**Keywords:** Hybrid imaging, Survey, PET/CT, Training, Procedures

## Abstract

**Objectives:**

To assess the current state of hybrid imaging in Europe with respect to operations, reading and reporting, as well as qualification and training.

**Methods:**

The first survey (LOCAL) was sent to the heads of the departments of radiology and nuclear medicine in Europe in 2017, including 15 questions regarding the organisation of hybrid imaging operations, reporting strategies for PET/CT and the existence of relevant training programmes. The second survey (NATIONAL) consisted of 10 questions and was directed to the national ministries of health of 37 European countries addressing combined training options in radiology and nuclear medicine.

**Results:**

In the LOCAL survey, 61 valid responses from 26 European countries were received. In almost half of the institutions, hybrid imaging was performed within a single department, mainly in nuclear medicine departments (31%). In half of the centres (51%), PET/CT reports were performed jointly, while in 20% of the centres, reporting was performed by nuclear medicine physicians. Radiologists were responsible for presenting hybrid imaging results in clinical boards in 34% of responding sites. Integrated hybrid imaging training was available in 41% sites. In the NATIONAL survey, responses from 34 countries were received and demonstrated a heterogeneous landscape of official training possibilities in radiology and nuclear medicine with limited opportunities for additional qualifications in hybrid imaging.

**Conclusions:**

The results of these surveys demonstrate a notable heterogeneity in the current practice of hybrid imaging throughout Europe. This heterogeneity exists despite the general consensus that strong professional cooperation is required in order to ensure high clinical quality and to strengthen the clinical role of hybrid imaging.

## Key points

- Hybrid imaging practice in Europe is notably heterogeneous.
- Integrated, interdisciplinary PET/CT reporting is not implemented ubiquitously.
- Training programmes and qualification options in hybrid imaging are limited in Europe.
- Further harmonisation of operation and training in hybrid imaging is necessary on a European, regional and institutional level in order to ensure high clinical quality and to strengthen the clinical role of hybrid imaging.

## Introduction

Over the past two decades, hybrid imaging has evolved into a diagnostic standard in oncological imaging. The quality of structural and functional information provided by hybrid imaging modalities in vivo is unparalleled and opens perspectives for highly accurate diagnostics in the era of precision medicine. Despite controversies concerning distinct indications and questions of reimbursement, the use of hybrid imaging is expected to grow considerably in the future, particularly in view of the development of highly specific radiotracers [1].

The potential of hybrid imaging modalities—PET/CT [2], PET/MR [3] and SPECT/CT [4]—comes with a high degree of complexity with respect to both technical and clinical aspects. Furthermore, combining any two imaging modalities does not only affect engineers but imaging professionals alike [5]. While the technical aspects of hybrid imaging have been resolved to a high degree, its operating procedures are still under debate. These debates evolve around essential aspects, such as the use of standardised imaging protocols, diagnostic or organ localising CT, the adoption of an efficient image analysis and the pursuit of standard reporting strategies, as well as the need for integrated training and education options.

It appears that different positions concerning these questions depend to a certain degree on the individual background with the line of disagreement frequently running along the interface of radiologists and nuclear medicine specialists [6]. This may come as a surprise in light of existing statements and guidelines by the respective professional associations that demonstrate a high degree of a priori agreement on the central aspects of hybrid imaging [7, 8]. Finally, there is a principle understanding that hybrid imaging should exist as an integrated discipline in its own right for the sake of an increased diagnostic quality and, above all, for the benefit of the examined patients [7].

However, it is not clear how the goal of integrated hybrid imaging can be reached in more general terms. In order to better understand the potential roadblocks towards an updated training curriculum that includes hybrid imaging, it is essential to assess the current state and real-life conditions of performing hybrid imaging and then to align on cross-specialty strategies and future directions.

Here, we assess the state of affairs of hybrid imaging in Europe with respect to the procedures of operation, reading and reporting as well as qualification and training of the imaging experts. We expect a better understanding of any relevant variations in the recognition of hybrid imaging across Europe.

## Materials and methods

### Surveys

As an initiative of the European Society of Hybrid, Molecular and Translational Imaging (ESHI^MT^), two surveys were conducted in 2017 to address the procedures of hybrid imaging in Europe.

The first survey (LOCAL), consisting of 15 questions, was designed as a web-based electronic questionnaire using the “Google Forms” (Table 1). Heads of the departments of radiology or nuclear medicine were invited to participate. The names and contact information were obtained from the ESR list of heads and deans of radiology departments and institutes in Europe. The invitation was delivered by e-mail on Sep. 27, 2017, a reminder was sent on Oct. 27 and Nov. 24, 2017. Response collection was closed on Nov. 27, 2017.

**Table 1:** Questions of the LOCAL survey

Q1	What hybrid imaging procedures are performed at your hospital?
Q2	Where is the hybrid imaging facility located in your institute?
Q3	Who is responsible for the organization of your hybrid imaging facility (e.g. for the definition of imaging protocols)?
Q4	Who operates hybrid imaging scanners at your hospital?
Q5	How do you manage the injection of iodinated contrast medium in PET/CT?
Q6	Who performs the examination at your department?
Q7	Who takes care of reporting CT images in a PET/CT exam?
Q8	How do you report hybrid imaging procedures at your hospital?
Q9	Do you have a joint training programme for hybrid imaging in cooperation with nuclear medicine?
Q10	If no, would you support a joint training programme if nuclear medicine physicians did so too?
Q11	How are residents trained in your department with respect to hybrid imaging modalities?
Q12	Who presents hybrid imaging results to referring physicians in clinical boards (e.g. tumour boards)?
Q13	My country
Q14	My institution
Q15	How many PET/CT examinations does your institution acquire annually?

The second survey (NATIONAL) was directed by e-mail to the ministries of health of 37 member and observer countries of the Standing Committee of European Doctors (www.cpme.eu) and addressed the status of combined training options for the specialties of radiology and nuclear medicine in these countries. The questionnaire consisted of 10 questions (Table 2). The initial call was sent on Mar. 20, 2017, and a reminder was sent on Apr. 6, 2017. This survey was closed on Oct. 31, 2017.

**Table 2:** Questions of the NATIONAL survey

Q1	Country and name of national health office
Q2	Contact information
Q3	Required months of training to obtain a license in radiology
Q4	How many months of nuclear medicine training can be part of training in radiology?
Q5	Required months of training to obtain a license in nuclear medicine
Q6	How many months of radiology training can be part of training in nuclear medicine?
Q7	Is it possible to obtain both national licenses?
Q8	Number of licenses in your country in radiology and nuclear medicine
Q9	Are there partial qualifications for either radiologists of nuclear medicine physicians, e.g. PET qualification for radiologists or CT qualification for nuclear medicine physicians?
Q10	Additional comments

### Data analysis

The survey results are presented in a descriptive statistical manner using bar charts and pie charts for the purpose of visualisation. Proportions are expressed as percentage of the total number of replies.

## Results

### LOCAL survey

In total, 1361 invitations to participate in this survey were sent by e-mail. In 131 cases (10%), non-delivery notifications were received. In total, 61 responses (5%) from 26 countries were received. Most responses were from Spain (*n* = 7), Sweden (*n* = 5), the Netherlands (*n* = 5), Austria (*n* = 5) and Germany (*n* = 5). The majority of responses (82%) were received from university hospitals, and 13% of responses were received from public hospitals. The responding sites reported PET/CT and SPECT/CT to be their main hybrid imaging modalities with availabilities of 87% and 84%, respectively. PET/MR was available in 29% of the responding sites. About half of the sites (51%) performed more than 2000 PET/CT exams per year while 28% performed less than 1000 examinations per year.

**Fig. 1:**
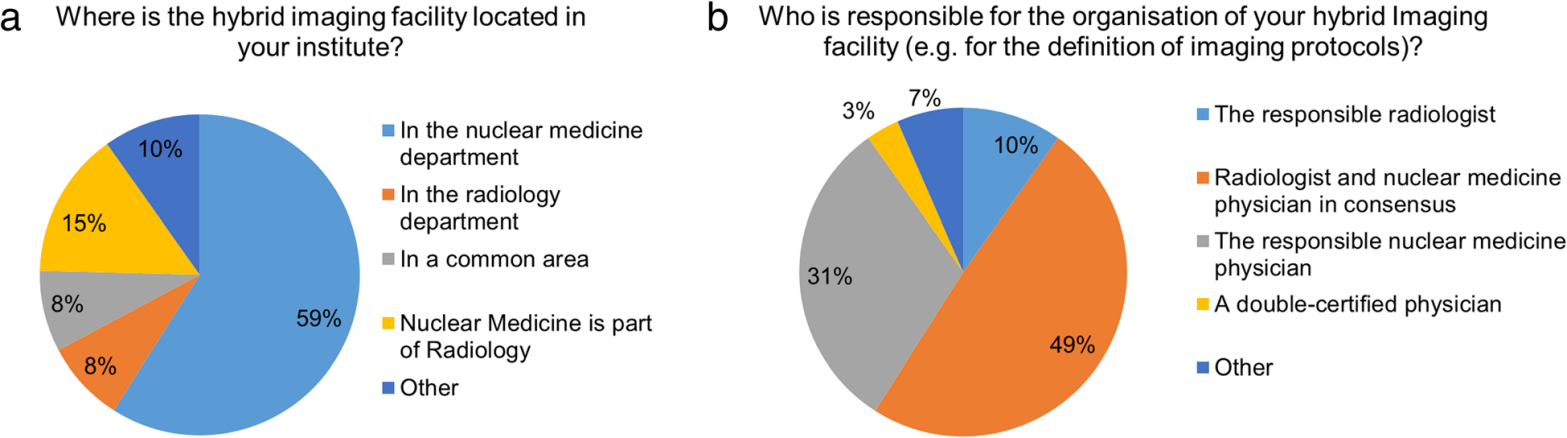
(**a**) Location of and (**b**) responsibility for hybrid imaging modalities

The majority of sites operated hybrid imaging systems in nuclear medicine departments (59%). Key responsibilities for hybrid imaging, including protocol definition, were in the hands of nuclear medicine physicians in 31% of sites (Fig. 1). In half of the sites (49%) responsibilities for hybrid imaging were shared (Fig. 1b). Hybrid systems were operated by nuclear medicine radiographers in the majority of cases (57%) and in 23% by radiographers from radiology and nuclear medicine together. In 90% of the departments, the application of intravenous CT contrast agents was part of routine PET/CT imaging protocols.

**Fig. 2:**
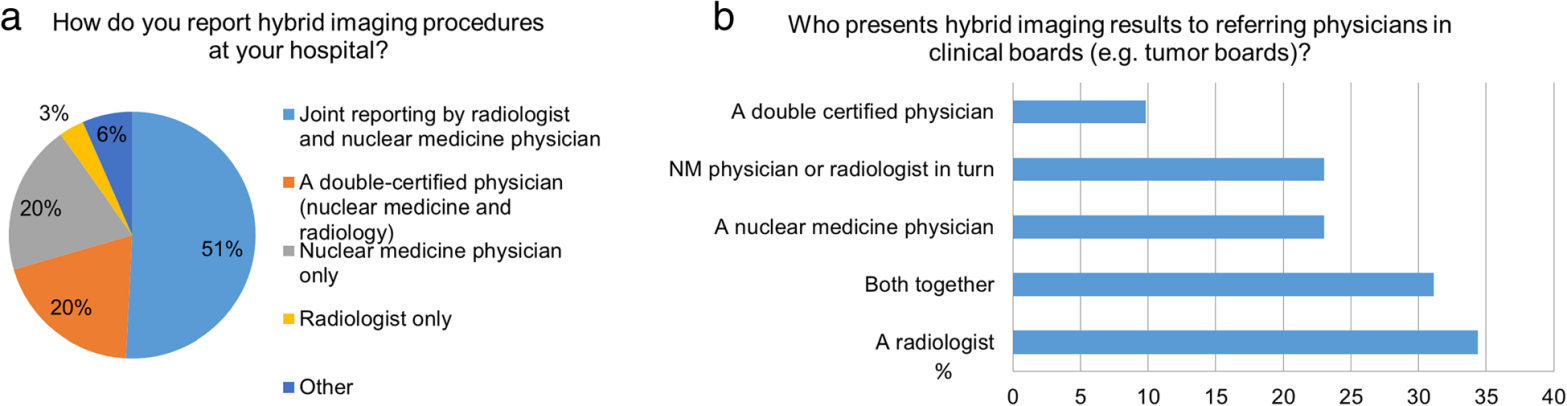
(**a**) Reporting and (**b**) presenting of hybrid imaging results

Responses for PET/CT reporting strategies were heterogeneous (Fig. 2). In about half (51%) of the participating sites, PET/CT reports were composed jointly by a radiologist and a nuclear medicine physician. In 20% of sites, a dual-certified imaging expert was responsible for PET/CT reporting; in another 20% of sites, PET/CT reporting was performed solely by a nuclear medicine physician. In contrast, the presentation of hybrid imaging results in tumour boards was more frequently performed by radiologists alone (34%) than by a nuclear medicine physician alone (23%) (Fig. 2b).

**Fig. 3:**
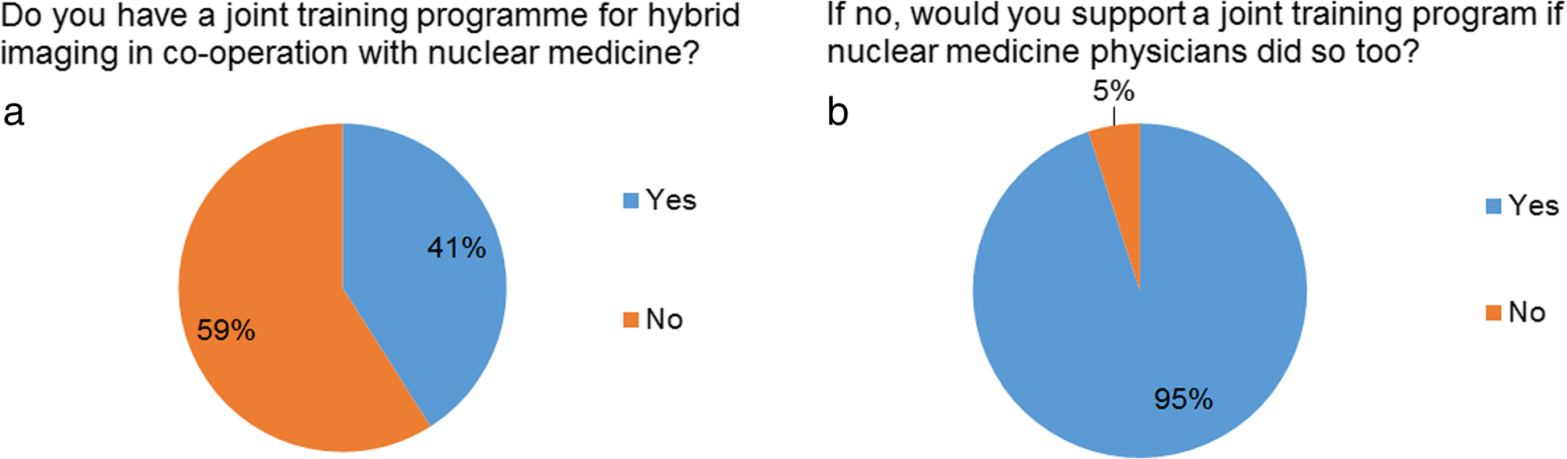
(**a**) Existence of and (**b**) support for joint training programmes

Only 41% of the respondents reported to offer a joint training programme in cooperation between radiology and nuclear medicine (Fig. 3a). Still, the majority of sites (95%) without locally available joint training would support the implementation of such a programme (Fig. 3b).

### NATIONAL survey

In total, replies from 34 of the 37 addressed ministries were received (response rate of 92%). The ratio of licenses in radiology and nuclear medicine was highly variable among the participating countries ranging from 4:1 to 49:1 (median 9.5:1).

In most countries (28/34, 82%), the duration of specialty training in radiology was 60 months (range from 48 months in five countries to 72 months in one country). The duration of nuclear medicine training was variable (12–60 months). In three countries, nuclear medicine did not exist as a separate specialty.

The duration of possible training periods in nuclear medicine as part of radiology programmes varied widely from 1–27 months (median 3 months) among the responding countries.

In only six countries, additional partial qualifications (e.g. for PET or CT) existed in nuclear medicine for radiologists or in radiology for nuclear medicine physicians. In 21 of 34 (62%) countries, dual certification in radiology and nuclear medicine is possible.

## Discussion

We explored the state of affairs for hybrid imaging in Europe in late 2017 using a web-based survey and direct questionnaires. Based on the responses, we observed a heterogeneous picture regarding central aspects of operation, reporting and training in multi-modality imaging. Despite the attempt to reach a wide number of participants throughout Europe, the response rate was only 5% in the LOCAL survey, thus limiting the generalizability of the results. Furthermore, the relatively high number of responding university sites should be noted, which may be seen as introducing a bias; on the other hand, these sites take a stronger inherent interest in responding to surveys as this. An additional source of potential bias is the fact that primarily heads of radiologist departments were addressed in the LOCAL study; heads of nuclear medicine departments were not specifically contacted due to the lack of available address database access. In the NATIONAL survey, we received responses from a wide range of European countries providing a representative picture especially with respect to training in hybrid imaging.

Most of the hybrid imaging systems are located in nuclear medicine departments with a significant number of these systems being operated by nuclear medicine specialists alone. This has likely historical reasons and can also be explained in part by the necessary measures for radiation protection. Our results reveal a high proportion of PET/CT sites that use intravenous CT contrast agents as also recommended by international guidelines [9]. Compared to the previously reported numbers, this is a substantial increase [10].

Joint PET/CT reporting, by complementary diagnostic experts, was performed at half of the responding sites. While this fraction has increased from a previous survey [10], it is still far away from a broad implementation of integrated reporting. Our survey indicates that still about 20% of reports are generated by a nuclear medicine physician alone. This result may be biased considering that we received responses mainly from university hospitals that may tend towards higher integration of PET/CT reporting. PET/CT reporting by a dual-board-certified imaging specialist was performed at 20% of responding sites. While the installation of single qualified readers may be the most efficient way of reading and reporting of hybrid imaging studies, this approach, however, would mandate broadly available training programmes.

In contrast, the presentation of hybrid imaging results as part of tumour boards is mainly a domain of radiologists [11]. The reasons for this observed imbalance of reading preference and external communication are not entirely clear but may originate from a traditionally stronger and wider integration of radiology experts in tumour boards and clinical review meetings. At the same time, these observations call for a solid training of imaging experts explaining hybrid imaging findings to treating physicians.

The observed heterogeneity in the adoption and operations of hybrid imaging is likely related to the organisation of hybrid imaging training. Integrated training curricula—although strongly recommended by the involved associations [7, 8]—are available only a very small number of hybrid imaging sites. Here, the proportion of integrated training sites may be overestimated in view of the relatively high fraction of responses received from university departments. Most importantly, we found that almost all participating sites would support a joint curriculum in hybrid imaging [12]. These observations are in concordance with the results of a recent survey among trainees in hybrid imaging that reported a lack of educational opportunities and a high demand for integrated training curricula [13]. Thus, the question arises why these curricula are not available at most sites despite the known need.

The heterogeneous landscape of official programmes in European countries may be one of the factors impeding the wide implementation of integrated training for hybrid imaging, as attested by the feedback from the NATIONAL survey. Only a minority of countries in Europe provide possibilities for additional qualifications in hybrid imaging for radiologists or nuclear medicine physicians. We can only speculate that professional political interests and divergent regional regulations inhibit faster progress [14]. Furthermore, differences in economic situations and divergent numbers of trained radiologists and nuclear medicine physicians may also contribute to the observed landscape. Nonetheless, positive examples for consistent integration of imaging specialties already exist in Europe [15] and could serve as the basis for subsequent training schemes elsewhere.

Compared to a previous survey on the practice of hybrid imaging in Europe performed by the European Society of Radiology and the European Association of Nuclear Medicine in 2009 [12], the described heterogeneity in practice of multi-modality imaging has still not been resolved.

It remains to be seen whether hybrid imaging should be regarded as a subspecialty of medical imaging by itself or rather as a collection of imaging methods that should be integrated into existing subspecialties. This discussion is ongoing with frequently extreme perspective on either end of the professional spectrum.

### Conclusion

The recent surveys among imaging professionals in Europe attest to a persistent heterogeneity in performing hybrid imaging examinations, in particular in regard to workflows, reporting and training. This heterogeneity exists despite repeated past cross-specialty statements that further integration of training procedures is required to make optimum use of hybrid imaging systems and, most importantly, for the benefit of the patients. In order to achieve this goal, a close and trusting cooperation between radiology and nuclear medicine and further harmonisation are necessary on a European, regional and institutional level. Also, a quality centric discussion about the necessary qualifications for reporting hybrid imaging studies should be initiated.
